# Murine Alox8 versus the human ALOX15B ortholog: differences and similarities

**DOI:** 10.1007/s00424-024-02961-w

**Published:** 2024-04-19

**Authors:** Megan A. Palmer, Yvonne Benatzy, Bernhard Brüne

**Affiliations:** 1 Institute of Biochemistry I, Faculty of Medicine, Goethe University Frankfurt, Theodor-Stern-Kai 7, 60590 Frankfurt, Germany; 2 Frankfurt Cancer Institute, Goethe University Frankfurt, Frankfurt, Germany; 3 Fraunhofer Institute for Translational Medicine and Pharmacology ITMP, Frankfurt, Germany; 4 German Cancer Consortium (DKTK), Partner Site Frankfurt, Frankfurt, Germany

**Keywords:** Cholesterol, Lipid peroxidation, Lipoxygenase, Oxylipins, Polyunsaturated fatty acids

## Abstract

Human arachidonate 15-lipoxygenase type B is a lipoxygenase that catalyzes the peroxidation of arachidonic acid at carbon-15. The corresponding murine ortholog however has 8-lipoxygenase activity. Both enzymes oxygenate polyunsaturated fatty acids in *S*-chirality with singular reaction specificity, although they generate a different product pattern. Furthermore, while both enzymes utilize both esterified fatty acids and fatty acid hydro(pero)xides as substrates, they differ with respect to the orientation of the fatty acid in their substrate-binding pocket. While ALOX15B accepts the fatty acid “tail-first,” Alox8 oxygenates the free fatty acid with its “head-first.” These differences in substrate orientation and thus in regio- and stereospecificity are thought to be determined by distinct amino acid residues. Towards their biological function, both enzymes share a commonality in regulating cholesterol homeostasis in macrophages, and Alox8 knockdown is associated with reduced atherosclerosis in mice. Additional roles have been linked to lung inflammation along with tumor suppressor activity. This review focuses on the current knowledge of the enzymatic activity of human ALOX15B and murine Alox8, along with their association with diseases.

## Introduction

Lipoxygenases (LOXs) are iron-containing dioxygenases that catalyze the hydroperoxidation of polyenoic fatty acids with distinct positional specificity and stereospecificity. Although the biochemical properties are well studied, the biological roles of the diverse LOX enzymes are still under investigation. The human genome involves six functional arachidonic lipoxygenase (*ALOX)* genes (*ALOX5*, *ALOX15*, *ALOX15B*, *ALOX12*, *ALOX12B*, and *ALOXE3*). Of these, all, except *ALOX5*, have been mapped to a joint gene cluster on chromosome 17 [60]. In mice, six of the seven genes encoding for *Alox5*, *Alox15*, *Alox8*, *Alox12*, *Alox12b*, *Aloxe3*, and *Aloxe12* (a corrupted pseudogene in human) are located in syntenic regions in a common gene cluster on chromosome 11 [27]. Similarly to the human genome, *Alox5* is located on a separate chromosome [18, 73].

The position of oxygen insertion into the carbon backbone of arachidonic acid (AA) determines the LOX nomenclature. Initially described in 1988, human leukocyte and reticulocyte 15*S*-lipoxygenase (15-LOX-1 or ALOX15) was the first human LOX identified with AA carbon-15 lipoxygenating properties [98, 99]. However, although ALOX15 forms mainly 15*S*-hydroperoxyeicosatetraenoic acid (HpETE), its dual-reaction specificity was demonstrated by the detection of its second but less formed product 12*S*-HpETE, generated from AA carbon-12 oxygenation, which initially has been described for the rabbit enzyme [62] and later for human ALOX15 [59, 63]. In 1997, a second 15-lipoxygenase called 15-LOX-2 or ALOX15B was identified with singular-reaction specificity, exclusively forming 15*S*-HpETE [11, 63]. In the same year, the group around Alan R. Brash also described its murine ortholog [47], a decade after the first detection of 8-hydreoxyeicosatetraenoic acid (HETE) in phorbol ester–treated mouse skin [36] and 6 years after the first mention of the epidermal mouse 8-lipoxygenase [28]. The murine AA carbon-8 oxygenating enzyme shares a high degree (78%) in amino acid conservation and hence a common ancestor with human ALOX15B [1] but differs drastically in regiospecificity, i.e., the site of oxygen insertion, as well as stereospecificity, i.e., hydrogen abstraction [47]. In contrast to human ALOX15B, the murine enzyme oxygenates AA to exclusively 8*S*-HpETE and thus was coined 8*S*-lipoxygenase [47]. Referring to the previously annotated human gene *ALOX15B* [29], the gene coding for the mouse 8*S*-lipoxygenase was thus named *Alox15b*; however, the official nomenclature is *Alox8*, which will be used for the remainder of this review.

## LOX-catalyzed substrate oxygenation

**Fig. 1:**
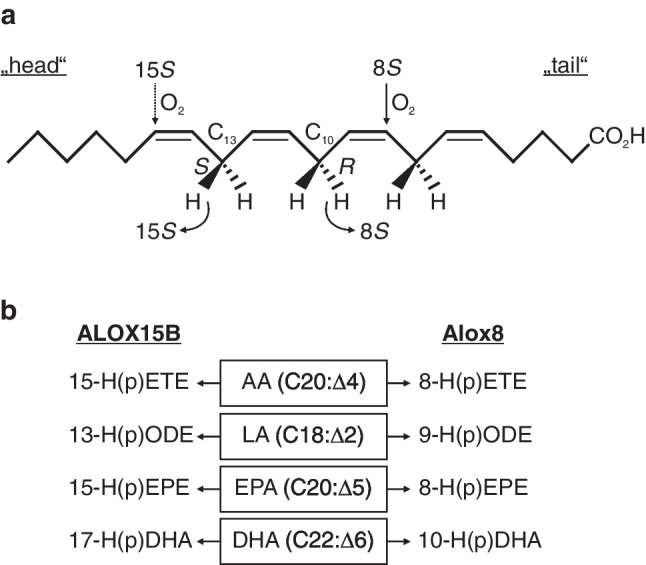
PUFA oxygenation by human ALOX15B and mouse Alox8. **a** Stereo- and regioselective reaction specificities of hydrogen abstraction (bended arrow) and oxygen insertion (solid arrow) of 15-lipoxygenating ALOX15B and 8-lipoxygenating Alox8 exemplified with arachidonic acid (AA). Solid arrows above AA show the site of oxygen insertion from the upper face, whereas dotted arrows indicate oxygen introduction from below. **b** Oxygenation products of AA, linoleic acid (LA), eicosapentaenoic acid (EPA), and docosahexaenoic acid (DHA) catalyzed by ALOX15B (left) and Alox8 (right). The resulting hydroperoxides can undergo reduction to the respective lipid hydroxides. H(p)ETE, hydro(pero)xy eicosatetraenoic acid; H(p)ODE, hydro(pero)xy octadecadienoic acid; H(p)EPE, hydro(pero)xy eicosapentaenoic acid; H(p)DHA, hydro(pero)xy docosahexaenoic acid

The widely accepted mechanism of LOX-catalyzed fatty acid peroxidation has recently been summarized for ALOX15B-mediated AA oxygenation [4]. In short, via their non-heme iron, LOXs catalyze the stereo- and regiospecific abstraction of a bis-allylic hydrogen from a CH2 methylene of a *cis*,*cis*-1,4-pentadiene. Upon rearrangement of the resulting radical, oxygen is introduced antarafacially to the site of hydrogen abstraction. Thereby, head-to-tail orientation of the substrate as well as the entry depth into the active site determines the enzyme’s regiospecificity. Further, the abstraction of the pro-*S* or pro-*R* hydrogen along with oxygenation of the proximal or distal end of the pentadiene determines the enzyme’s stereospecificity [76] (Fig. 1a). The resulting lipid hydroperoxides are subsequently reduced to the respective hydroxides by glutathione peroxidases [22], of which glutathione peroxidase 4 is the only one that reduces peroxidized complex phospholipids and cholesterol esters, also when incorporated into membranes [71].

### ALOX15B and Alox8-mediated substrate peroxidation

The ALOX15B-catalyzed oxygenation of the omega-6 (ω-6) polyunsaturated fatty acid (PUFA) AA (C20:Δ4) at carbon-15 to 15*S*-HETE was reported along with its first description [11]. However, further studies confirmed its singular reaction specificity also with ω-6 linoleic acid (LA) (C18:Δ2) as well as the less abundant ω-3 PUFAs eicosapentaenoic acid (EPA) (C20:Δ5) and docosahexaenoic acid (DHA) (C22:Δ6) [63]. ALOX15B-catalyzed oxygenation of LA results in the formation of 13*S*-hydroxy octadecadienoic acid (HODE); EPA is formed to 15*S*-hydroxy eicosapentaenoic acid (HEPE) and DHA to 17*S*-hydroxy docosahexaenoic acid (HDHA) [63] (Fig. 1b). Using purified, recombinant enzyme preparations, varying substrate preferences have been reported for ALOX15B. Whereas some groups reported a preferred oxygenation of AA compared to LA [12, 63], other groups described contrary results [55, 116]. Additionally, while using the recombinant enzyme DHA was found as the most preferred substrate compared to AA, LA, and EPA [63], cellular studies in primary human macrophages revealed higher product formation from AA and EPA than from DHA peroxidation [19]. Even phospholipid-esterified PUFAs, including very large esters of phosphatidylcholine, were found to be oxygenated by ALOX15B with singular positional specificity [6]. However, ALOX15B activity with free and esterified substrates is markedly different. Recapitulating data reported by Bender et al. [6], using recombinant ALOX15B and free AA, *k*_cat_/*K*_m_ appeared to be 0.264 ± 0.121 s^−1^ μm^−1^, whereas for the reaction of ALOX15B with AA-ethyl ester, *k*_cat_/*K*_m_ was found to be only 0.0464 ± 0.0084 s^−1^ μm^−1^.

Regioselective oxygenation products of ALOX15B’s murine ortholog Alox8 were identified as well; however, in contrast to ALOX15B, free AA is peroxidized to 8*S*-HETE [47] and LA is peroxidized to 9*S*-HODE, whereby the reaction with LA was found to be around threefold lower compared with AA [47]. Alox8-mediated oxygenation of DHA gives rise to 10-HDHA, while EPA is metabolized to 8*S*-HEPE [96] (Fig. 1b). In Alox8-expressing *E. coli* cells, the substrate preference of AA was reported in addition to oxygenation of EPA being greater than DHA [96]. Additionally, enzymatic studies with recombinant Alox8 accepted both α- and γ-linolenic acids as substrates [83], along with the synthesis of 8,9-leukotriene A4 from 5-HETE [53, 83, 95]. Moreover, both mammalian orthologs were found to oxygenate lipid hydro(pero)xides. For example, Alox8 oxygenates 15-HETE to 15,8-diHETE in vitro and ALOX15B converts 8-HETE to the double-oxygenation product 8,15-diHETE [50]. In addition, ALOX15B peroxidizes ALOX5-derived 5-H(p)ETE to 5,15-diH(p)ETE [33, 79], which can be enzymatically processed via ALOX15 and ALOX12, but not via ALOX15B [33], to lipoxin (LX) B4 [34]. Although it has been reported that purified ALOX5 cannot use exogenous 5,15-diH(p)ETE for LX formation in vitro, conversion of 15-HpETE to LXA4 by ALOX5 indicated the oxygenation of 5,15-diH(p)ETE to LXA4 in situ [34]. However, further studies demonstrated the dependence on arachidonate 5-lipoxygenase activating protein, short ALOX5AP or FLAP, for the formation of LXA4 [65]. Cellular studies using human leukocytes revealed very low formation of both di-hydroxylated epoxide precursors and tri-hydroxylated oxygenation products, including LXs [49]. Along these lines, concerns about the physiological relevance and reliable detection of di- and tri-hydroxylated metabolites emerged as a current topic of debate [49, 90].

Of note, the discrepancies in product formation between mammalian LOX orthologs are not limited to ALOX15B and Alox8. Also the murine ortholog of ALOX15 differs in product formation from its human counterpart as it performs predominantly AA 12-lipoxygenating activity and thus, apart from Alox15, is called 12/15-LOX [1, 26]. However, whereas the ALOX15- and Alox15-mediated oxygenation of α-linolenic acid and EPA as well as of LA leads to identical oxygenation products from both enzymes [1, 63], ALOX15B and Alox8 also differ in this respect, as stated above. While the different reaction specificities of human ALOX15 and its murine counterpart can be evolutionary explained [1, 60], this is not applicable to ALOX15B and Alox8 reaction specificities. In most mammalian ALOX15B orthologs, the amino acid determinants that cause regiospecific substrate peroxidation are similar to those of human ALOX15B, as explained later in this review, as they all contain Asp at position 603; however, Alox8 is an exception as it carries a Tyr at this position [84]. Based on the reported differences in oxygenation products, varying intra- and transcellular processes are assumed for both human ALOX15B and murine Alox8. Although Lehmann et al. [66] reported the simultaneous increase of 8-HETE in parallel with 9*S*-HODE in murine skin papilloma, from which the latter can be distinguished from cyclooxygenase-derived 9*R*-HODE by its chirality [38], auto-oxidation products produce racemic mixtures, forming both 9*S* and 9*R* enantiomers. Therefore, the origin of oxygenation products should always be critically evaluated, and the amount of oxygenation products derived from spontaneous oxidation should not be underestimated.

### Stereospecificity of ALOX15B- and Alox8-catalyzed PUFA oxygenation

Long-term molecular dynamics simulations indicated the abstraction of the pro-*S* hydrogen of carbon-13 from AA by ALOX15B [104], whereas an attack at the pro-*R* hydrogen of carbon-10 was proposed for Alox8 [10, 41, 48] (Fig. 1a). According to Coffa and Brash [15], the LOX stereoselective oxygenation of PUFAs into either the *S*- or *R*-enantiomer of the resulting hydroperoxide is determined by a single amino acid residue in the LOX active center. Mutation of Ala to Gly of murine Alox8 (8*S*-LOX) as well as human ALOX15B (15*S*-LOX) altered their *S*-stereoselective peroxidation into mainly the respective *R*-enantiomers (Alox8, 8*S*-HETE into 12*R*-HETE and 8*S*-HETE in a 1.8:1 ratio; ALOX15B, 15*S*-HETE into 11*R*-HETE and 15*S*-HETE in a 1.5:1 ratio). Vice versa, in human 12*R*-LOX and coral 8*R*-LOX, the mutation of Gly to Ala favored the formation of the corresponding *S*-oxygenation products (12*R*-LOX, 12*R*-HETE into 8*S*-HETE and 12*R*-HETE in a ratio of 1.4:1; 8*R*-LOX, 8*R*-HETE into almost exclusively 12*S*-HETE). Yet, the Ala-versus-Gly concept is not applicable to all ALOX isoforms. Although zebrafish LOX1 catalyzes almost exclusively 12*S*-lipoxygenation from AA, it contains a Gly at this critical position. While Jansen et al. (2011) found massive alterations concerning reaction specificity and increased *R*-conformation upon Gly-to-Ala exchange in human ALOX15B and platelet ALOX12, only minor alterations in reaction specificity were observed for murine Alox5 as well as human ALOX15 and its mouse, rabbit, rhesus monkey, and orangutan orthologs. Moreover, only 10–30% of the products found were *R*-HETE, whereas the majority was peroxidized to *S*-HETE [45].

### Oxygenation of esterified substrate by ALOX15B and Alox8

In contrast to ALOX15B [6, 15], Alox8’s ability to oxygenate esterified substrates was undetermined for a long time. As addressed in the following section, inverted head-to-tail substrate orientation of Alox8 was assumed to prevent the entry of the bulky carboxyl-end (“head”) into its substrate binding pocket and hence the entry of esterified substrates. However, using AA-containing phospholipid liposomes [50] and AA-containing phospholipids in nanodiscs [6], it was demonstrated that the bulky phospholipid head group forces Alox8 towards a tail-first entry, provoking the formation of 15-HETE by both ALOX15B orthologs. These nanodiscs moreover contained dihomo-γ-linolenic acid (DGLA), resulting in the formation of 15-hydroxy eicosatrienoic acid (HeTrE) from both enzymes. Here, the ALOX15B-catalyzed formation of 15-HETE was 6–10 × higher than Alox8’s, whereby Alox8 oxygenated preferentially the less abundant DGLA. However, as a free substrate, AA was preferred over DGLA by Alox8 and slightly also by ALOX15B [6]. Using mitochondrial membranes or LA-containing liposomes, however, different reaction products of ALOX15B and Alox8 were detected. Here, ALOX15B catalyzed the formation of 15-HETE and 13-HODE, whereas Alox8 formed mainly 9-HODE and only minor amounts of AA oxygenation products. As indicated by the authors, additional work is required to answer whether the reaction specificities may differ between cellular membranes or different lipid-protein complexes [50]. Based on their results with phospholipid-esterified AA, Bender et al. [6] points out possible implications of an in vivo 15-LOX activity of Alox8, but the more recent data from Kakularam et al. [50] make a general in vivo 15-LOX activity of Alox8 with esterified substrates unlikely. However, besides free versus esterified substrates, along with the phospholipid headgroup, substrate concentration also impacts the product pattern of Alox8. Whereas at high EPA concentrations 8-HEPE is the only product formed by Alox8 [6], at low substrate concentrations Alox8 also generates moderate amounts of 15-HEPE [50]. As indicated by the authors of the study, this should be considered when oxygenation products from mouse cells, tissues, and body fluids are analyzed [50]. In general, it is important to acknowledge the limitations in scope and physiological significance of data generated using enzyme preparations and overexpression systems.

### Regiospecificity of ALOX15B- and Alox8-catalyzed PUFA oxygenation

The regiospecificity of substrate oxygenation and the differences in oxygenation products between ALOX orthologs have been explained by biochemical and structural properties of distinct amino acid residues. For mammalian ALOX15 orthologs, the “Triad Concept” was developed, involving three amino acid clusters, that determine the orientation of substrate entry as well as the enzymes’ positional specificity [7, 8, 100]. In detail, the side chain geometry of the amino acid determinants allows the deepness of fatty acid penetration into the hydrophobic substrate-binding pocket, thus placing the respective fatty acid bis-allylic methylenes in the closest proximity to the reactive non-heme iron [61]. However, this concept was found to be not applicable to ALOX15B-mediated substrate oxygenation [112].

Like the “Triad Concept” that explains ALOX15-dependent regiospecificity [7, 8, 43], positional and structural determinants of ALOX15B- and Alox8-dependent substrate oxygenation have been described. Crystal structure analysis of LOXs with substrate mimetics [57, 57] as well as AA under anaerobic conditions [75] indicated the existence of a U-shaped substrate-binding cavity in which distinct amino acids determine the head-to-tail orientation of substrate entry as well as the final position in the active site [76]. For human ALOX15B, AA entry in a tail-first orientation has been suggested, as this positions the polyenoic fatty acid for oxygen insertion at carbon-15 [15, 57, 104]. Additionally, molecular docking simulations showed that hydrogen abstraction from carbon-13 in the tail-first orientation mediates the sterically preferred position for AA oxygenation by ALOX15B [50]. In contrast, the murine ortholog Alox8 accepts the AA entry with the carboxyl-end, which sterically favors the hydrogen abstraction from carbon-10 [50] and hence synthesizes 8-HpETE instead of 15-HpETE [12, 48, 50] (Fig. 1a).

Using in vitro mutagenesis studies of the C-terminal catalytic domain of murine Alox8 and human ALOX15B, the structural basis for the different positional specificities of mouse and human enzyme reactions has been explored [48]. In human ALOX15B, mutation of the catalytic center amino acids Asp^602^/Val^603^ to the corresponding amino acids of murine Alox8, Tyr^603^/His^604^ altered the oxygenation products from 15-HpETE towards mainly 8-HpETE and vice versa [48]. Further in vitro mutagenesis studies using humanized Alox8 demonstrated the change from carbon-8 oxygenation towards the respective carbon-15 oxygenation products with both AA and EPA, whereas the murinization of human ALOX15B changed the product pattern towards carbon-8 oxygenation products [50]. However, although working with C20 fatty acids, this was not the case for LA and DHA products. Humanization of Alox8 mediated the generation of the correct DHA products (17-HDHA), whereas murinization of ALOX15B yielded only minor amounts of 10-HDHA (the product of wild-type Alox8 with DHA as a substrate). In fact, the main product was 7-HDHA, which is usually produced by Alox5. Similarly, substitution of murine Tyr^603^/His^604^ towards human Asp^603^/Val^604^ yielded the expected 13-HODE from LA oxygenation, hence confirming the humanization. In contrary, murinization catalyzed the formation of a racemic product mixture of 13*S*- and 13*R*-HODE as well as 9*S*- and 9*R*-HODE, with a more pronounced formation of the respective *S*-enantiomers [50]. Since for Alox8 an inverse substrate entry with PUFA head-first has been suggested for AA, molecular docking calculations were performed to determine the orientation of EPA, DHA, and LA in the active sites of wild-type ALOX15B and Alox8 as well as their humanized and murinized mutants, respectively. In line with the previously reported AA tail-first substrate entry in human ALOX15B, molecular docking calculations indicated the tail-first entry of each of the selected PUFAs also for humanized Alox8. Furthermore, for murine Alox8 and murinized ALOX15B, a preferred head-first substrate entry was determined [50].

As already indicated by Jisaka et al. [48], further studies demonstrated the importance of the histidine regarding Alox8’s positional specificity and orientation of substrate entry in comparison to the 15-lipoxygenating human ortholog. Walther and colleagues [113] investigated the effect of pH-dependent changes on product specificity and found that the substrate orientation was altered at acidic and alkaline pH. At acidic pH, the protonation of the carboxylic group of the substrate fatty acid favored the interaction with the chargeable histidine in the active site of Alox8 and therewith an inverse head-to-tail substrate orientation. Entry with the carboxyl head-first promoted carbon-8 oxygenation of AA. Although over a wide range of pH 8-HpETE was found as the major oxygenation product of Alox8, at alkaline pH (≥ pH 9), deprotonation of the carboxyl-end as well as the enzyme’s histidine also enabled the formation of 15-HpETE [113]. Another study demonstrated that in contrast to human ALOX15B, which showed the highest EPA oxygenation rate at neutral pH (pH 7.4), its murine ortholog Alox8 exhibited the highest activity and 8-HpETE formation at pH 6.4 [50]. However, Walther et al. [113] also described that mutant Alox8, in which the His^604^ was replaced by Phe, still was able to oxygenate a dicarboxylic fatty acid, whereas the His^603^-containing ALOX15B mutant did not accept the dicarboxylic derivative of AA although the introduction of a His^603^ previously converted the exclusively 15-lipoxygenating enzyme into a major 8-lipoxygenating enzyme. Based on this discrepancy, the authors concluded that besides the presence of a chargeable histidine at the active site additional structural effects must account for the different positional specificities between the two mammalian ALOX15B orthologs. Jisaka and coauthors [48] hypothesized the interaction of the critical Tyr^603^/His^604^ amino acid pair with the enzyme’s helices and thus an implication in the formation of the substrate-binding pocket. Additionally, Walther et al. [113] pointed out that removal/addition of a histidine by mutagenesis may lead to a comprehensive restructuring of the substrate-binding pocket via an altered hydrogen bonding network as well as alterations in the amino acid pKa values along with changes in amino acid interactions.

## Investigations on mice with point mutations of Alox8 resulting in humanized reaction specificities

Based on the in vitro identification of Tyr^603^/His^604^ (human ALOX15B) and Asp^603^/Val^604^ (mouse Alox8) as critical amino acids whose mutual exchange mediates the conversion from ALOX15B towards Alox8 reaction specificity and vice versa, homozygous humanized *Alox8* knock-in mice were generated, which originally were declared as *Alox15b* knock-in (KI) mice [88, 89]. These mice exclusively express the AA 15-lipoxygenating double mutant of Alox8, instead of the wild-type Alox8. Upon substitution of Tyr^603^/His^604^ to Asp^603^/Val^604^, no formation of 8-HETE but increased 15-HETE levels were detected in ex vivo activity assays of PMA-treated mouse epidermis, hence supporting the previously generated in vitro mutagenesis data [88]. However, analysis of oxylipins in blood plasma of Alox8-humanized mice neither revealed an increase in AA-derived 15-HETE, EPA-derived 15-HEPE, DHA-derived 17-HDHA, nor a decrease in 8-HETE, 8-HEPE, and 10-HDHA when compared to outbred wild-type mice. Only 15-HeTrE, the human ALOX15B oxygenation product from DGLA, increased in mice with Alox8-humanized reaction specificities. However, no concomitant reduction in 8-HeTrE was detected. According to the authors, it remains to be explored how the subtle changes in plasma oxylipins are related to the small genetic manipulation [88]. In detail, the study exhibited that aged male mice with humanized Alox8 reaction specificities experienced a gender-specific growth arrest, which might be impacted by their significantly attenuated red blood cell parameters, including erythrocyte numbers, hematocrit, and hemoglobin [88].

Whereas so far, no further studies report a link of ALOX15B or Alox8 with those parameters, systemic inactivation of murine Alox15 also negatively affected erythropoiesis. In these *Alox15*^−/−^ mice, transgenic expression of human ALOX15 rescued the defective erythropoietic system and the impaired osmotic resistance of the erythrocytes [84]. In this context, also the transgenic introduction of ALOX15B in mice lacking Alox8 would help to clarify whether both enzymes mediate similar processes, although they produce very different oxygenation products.

A follow-up study in young mice analyzed the impact of Alox8 humanization regarding different inflammation models [89]. Female Alox8-humanized mice lost more body weight during the acute phase of dextran sodium sulfate–induced colitis. This was accompanied by a less rapid recovery during the resolution phase of inflammation. However, the histological analysis suggested no significant difference in the degree of intestinal inflammation between the genotypes as well as no differences in pro- and anti-inflammatory eicosanoids [89]. In general, analysis of colon tissue from untreated Alox8-humanized mice revealed no increase in 15-HETE, 15-HEPE, and 15-HETrE but a decrease in 8-HETE and 8-HTrETE levels [89]. It was discussed whether the lack of detection of elevated 15-hydroxy-PUFAs could be caused by high basal levels as well as their rapid metabolism to secondary products. The authors concluded that the induced colitis more severely compromised the intestinal water barrier of humanized Alox8 than of wild-type mice, without affecting the degree of inflammation. They also detected a contrasting impact of Alox8 humanization of different inflammation models. Whereas in experimental colitis Alox8-humanized mice were sensitized for colitis onset, the humanization partly protected from paw-edema inflammation, without affecting pain perception. Conversely, in human colonic mucosa, reduced ALOX15B levels were found in samples from ulcerative colitis patients in comparison to healthy controls [72]. Given the various and controversial links of ALOX15B to different inflammatory diseases [4], further studies are necessary to investigate the role of ALOX15B in inflammatory conditions. However, the data from Alox8-humanized mice support the previously reported ambivalent role of ALOX15B in inflammation.

Based on the reduced detection of Alox5-dependent oxygenation products in the colon, the authors suggested that Alox8 might impair the catalytic activity of Alox5 since Alox5-derived leukotrienes are established mediators in paw edema [89]. In contrast, in peripheral blood cells, no differences in Alox5 activity were reported between wild-type and *Alox15b* knock-in mice [88]. However, incubation of purified recombinant murinized ALOX15B with AA revealed the formation of 5*S*-HETE as a side product [50], which might indicate that Alox8 is capable of forming small amounts of 5-HETE. Therefore, reduced levels of 5-HETE could be due to a lack of intact Alox8 enzyme but independent of ALOX15B enzymatic activity.

Overall, based on the ex vivo analysis of skin oxylipins from PMA-treated mice, the in vitro–predicted switch in reaction specificity of wild-type Alox8 to oxygenation products of human ALOX15B was confirmed. While in wild-type mice about 20% of all products were identified as 8-HETE, *Alox15b*-knock-in mice formed 20% 15-HETE and less than 1% 8-HETE [50]. However, the humanization did not change plasma levels in the expected direction. Whether the degree of changes in oxylipins is related to the tissue/cell expression of Alox8 should be examined in further studies. Moreover, it should be addressed to what extent the humanization and not the loss of wild-type Alox8 activity causes the reported differences in bodyweight and inflammation as well as the above-mentioned effects on the erythropoietic system. Given that *Alox15b*-knock-in mice were compared with outbred wild-type mice, the reported effects could be due to both the shift in reaction specificities from the murine to the human enzyme as well as the loss of Alox8 activity and the absence of its metabolites, which are not compensated for by the humanized products. The impact of missing Alox8 activity in comparison to gained ALOX15B activity should be investigated in the future. However, with limited data available to date from Alox8-deficient mice, these studies provide a unique opportunity to gain insights into the biological function of human ALOX15B and mouse Alox8 in vivo.

## Tissue expression of Alox8 and ALOX15B

Alox8 was originally identified and first described in phorbol ester–treated mouse skin, where its expression greatly increased, particularly in the stratum granulosum [28, 39, 47]. This is supported by more recent studies, which report highest *Alox8* gene expression in mouse skin, but also in the lung [39, 88]. Similarly, its human ortholog ALOX15B is described in the skin and lung [11], various bronchial cell lines [55, 105], normal or immortalized normal keratinocytes and melanoma cells [105], and benign lung [31]. In contrast, low Alox8 expression levels were identified in the liver, kidney, and bone marrow [88]. Likewise, in tumor-associated macrophages of renal cell carcinomas [16] as well as in hepatocellular carcinoma cell lines [105], ALOX15B expression was detected. Additional gene expression analyses indicate low constitutive expression of *Alox8* in the colon and brain [39], and also ALOX15B was detected in freshly isolated enteric glial cells [80] and cell line–derived dopaminergic neurons [9]. While no Alox8 oxygenation products were detected in the prostate, ALOX15B expression is present [11]. Alox8 expression increased in murine brains following traumatic brain injury along with several other genes linked to ferroptosis [119]. This increase in expression was reduced following intermittent fasting.

Of note, investigations in skin of different mouse strains revealed major differences of both the constitutive and inducible expressions of Alox8 [20, 21, 47]. Whereas in both the skin of NMRI and black Swiss mice a low constitutive but strongly phorbol ester inducible Alox8 expression was seen, young SENCAR mice expressed *Alox8* constitutively at high levels but exhibited only low inducibility by phorbol esters [47]. Moreover, no phorbol ester–mediated induction of Alox8 activity was seen in the skin of C57BL/6 J mice at all [20]. These discrepancies are probably not limited to the skin. Therefore, comparability of data from different mouse strains and in particular the comparability with expression in human tissue is very limited and should be interpreted with caution.

It should be noted that many expression studies investigating Alox8 were performed in the 1990s with only semi-quantitative techniques (Northern blot and RT-PCR). Table 1 provides a detailed list of published tissue expression analysis for Alox8. Although some studies have used Western blotting to detect Alox8 protein, metabolite analysis provides a more useful tool in deciphering the catalytic activity of Alox8 in murine tissues. To date, endogenous 8-HETE or 9-HODE has only been reported in the skin [56], lung [3, 103, 110], and intestine [28, 30]. The skin remains the only tissue in which ex vivo activity assays of Alox8 have been performed with AA and LA [13, 20, 28]. Further analysis via mass spectrometry is needed from *Alox8*^−/−^ mice to ensure the enzyme is catalytically active in tissue with positive mRNA or protein expression.

**Table 1:** Tissue expression of Alox8

Tissue	Expression	Measurement	Method	Mouse strain	Antibody	Validation	Ref
Aorta	Positive	mRNA	qPCR	C57BL/6		shRNA	[70]
Aorta	Positive	Protein	IHC, WB	C57BL/6	Oxford antibodies (LX25)	shRNA	[70]
BMDM	M0, M1 and M2	Protein	Flow cytometry	C57BL/6 J	Santa Cruz (sc-271290)		[51]
BMDM	M0, M1 and M2	Protein	Western blot	C57BL/6 J	Santa Cruz (sc-67143)	siRNA knockdown in RAW 267.4 and EOC 20 cells	[51]
Bone marrow	< 4 × 10^4^/10^6^ GAPDH	mRNA	qPCR	Unknown			[88]
BMDM	Positive	mRNA	qPCR	C57BL/6		shRNA	[70]
BMDM	Positive	Protein	Western blot	C57BL/6	Oxford antibodies (LX25)	shRNA	[70]
Brain	Positive	mRNA	RT-PCR	NMRI			[39]
Brain	Positive	mRNA	Northern blot	C57BL/6			
Brain	Positive	mRNA	qPCR	C57BL/6N			[119]
Brain	Positive	Protein	Western blot	C57BL/6N	Protein tech (13,073–1-AP)		[119]
Brain	< 4 × 10^4^/10^6^ GAPDH	mRNA	qPCR	Unknown			[88]
Colon	Positive	mRNA	RT-PCR	NMRI			[39]
Colon	< 4 × 10^4^/10^6^ GAPDH	mRNA	qPCR	Unknown			
Forestomach	Low	mRNA	RT-PCR	NMRI			[39]
Heart	Negative	mRNA	Northern blot	C57BL/6			
Heart	< 4 × 10^4^/10^6^ GAPDH	mRNA	qPCR	Unknown			[88]
Intestine	Positive	mRNA	Nested PCR	C57BL/6Ntac			[52]
Kidney	Low?	mRNA	RT-PCR	NMRI			[39]
Kidney	Negative	mRNA	Northern blot	C57BL/6			[47]
Kidney	< 4 × 10^4^/10^6^ GAPDH	mRNA	qPCR	Unknown			[89]
Liver	Negative	mRNA	RT-PCR	NMRI			[39]
Liver	Negative	mRNA	Northern blot	C57BL/6			[47]
Liver	> 4 × 10^4^/10^6^ GAPDH	mRNA	qPCR	Unknown			[47]
Lung	Positive	mRNA	RT-PCR	C57BL/6N			[2]
Lung	Positive	Protein	Western blot	C57BL/6N	Santa Cruz (sc-271290)	Protein detected in KO mice	[2]
Lung	Positive	mRNA	RT-PCR	NMRI			[39]
Lung	Negative	mRNA	Northern blot	C57BL/6			[47]
Lung	> 4 × 10^5^/10^6^ GAPDH	mRNA	qPCR	Unknown			[88]
Muscle	< 4 × 10^4^/10^6^ GAPDH	mRNA	qPCR	Unknown			[88]
Platelets	Negative	mRNA	RT-PCR	NMRI			[39]
Prostate	Negative	Protein	IHC	Unknown	Oxford antibodies (LX25)		[106]
Reticulocytes	Negative	mRNA	RT-PCR	NMRI			[39]
Retina	Positive	Protein	Western blot	C57BL/6 J	abcam (ab23691)		[94]
Skeletal muscle	Negative	mRNA	Northern blot	C57BL/6			[47]
Skin	TPA treatment 4-48 h	mRNA	Northern blot	NMRI			[13]
Skin	6 h TPA, Papilloma	mRNA	RT-PCR	NMRI			[13]
Skin	Epidermis (TPA 0.5-48 h)	mRNA	RT-PCR	NMRI			[39]
Skin	Foot sole positive	mRNA	RT-PCR	NMRI			[39]
Skin	PMA 50 nmol 24 h	mRNA	Northern blot	Black Swiss			[47]
Skin	PMA 50 nmol 24 h	Protein	Western blot	Black Swiss	Polyclonal antibody raised against human ALOX15B		[47]
Skin	Elevated with TPA 50 nmol 24 h	Protein	IHC	Black Swiss			[47]
Skin	Acetone	Protein	IHC	Black Swiss			[47]
Skin	Constitutive in hair follicles	Protein	IHC	Black Swiss			[47]
Skin	Low level (9 h TPA)	mRNA	Northern blot	C57BL/6 J			[56]
Skin	Negative (9 h TPA)	Protein	Western blot	C57BL/6 J	[47]		[56]
Skin	Positive in differentiated layers. Elevated with 2 h TPA, Papilloma, decreased in Carcinoma	Protein	IHC	C57BL/6 J	[47]		[56]
Skin	Epidermis (TPA 4 h), Foot sole	mRNA	RT-PCR	Unknown			[58]
Skin	Epidermis (TPA 18 h), Foot sole	mRNA	Northern blot	Unknown			[58]
Skin	> 4 × 10^5^/10^6^ GAPDH	mRNA	qPCR	Unknown			[88]
Skin	*IκB-α*^−/−^	mRNA	Northern blot	C57BL/6			[91]
Skin	Negative in control	Protein	Western blot	C57BL/6	[47]		[91]
Skin	*IκB-α*^−/−^	Protein	Western blot	C57BL/6	[47]		[91]
Skin	Control and IMQ 6 days	mRNA	qPCR	FVB/N JC			[114]
Skin	Control and IMQ 6 days	Protein	Western blot	FVB/N JC	LSBio (LS-B1588-1)		[114]
Small intestine	Positive	mRNA	RT-PCR	NMRI			[39]
Spinal cord	Positive	mRNA	qPCR	C57BL/6			[122]
Spinal cord	Positive	Protein	Western blot	C57BL/6	Protein tech (13073–1-AP)		[122]
Spleen	Negative	mRNA	Northern blot	C57BL/6			[46]
Spleen	< 4 × 10^4^/10^6^ GAPDH	mRNA	qPCR	Unknown			[88]
Stomach	< 4 × 10^4^/10^6^ GAPDH	mRNA	qPCR	Unknown			[88]
Testis	Negative	mRNA	RT-PCR	NMRI			[39]
Testis	Negative	mRNA	Northern blot	C57BL/6			[46]
Testis	< 4 × 10^4^/10^6^ GAPDH	mRNA	qPCR	Unknown			[88]
Tongue	Negative	mRNA	RT-PCR	NMRI			[39]
Trachea	Negative	mRNA	RT-PCR	NMRI			[39]

*BMDM* Bone marrow-derived macrophages, *TPA* 12-O-tetradecanoylphorbol-13-acetate, *IMQ* Imiquimod, *IHC* Immunohistochemistry

## Alox8 and ALOX15B in disease

Recently, we reviewed the role of ALOX15B in the disease [4]. Therefore, the following sub-sections will focus on the research indicating the role of Alox8 in the disease and the comparison to what is known about human ALOX15B in diseases.

### A role for Alox8 and ALOX15B in lung inflammation

A previous study investigated *Alox8*^−/−^ mice in relation to influenza infections [2]. Although no differences were reported for body temperature, locomotion activity, or body weight prior to viral infection or infection in 3-month-old mice, impaired recovery was reported for 6-month-old mice. Furthermore, increased levels of inflammation were detected in infected 6-month-old *Alox8*^−/−^ mice via histological analysis and increased levels of cytokines chemokine (C-X-C motif) ligand 1 and interleukin (IL)-6 in the lungs. It should be noted that Western analysis of Alox8 revealed a low level of protein in the *Alox8*^−/−^ mice and that the enzymatic function was not investigated. Whether the band in the blot represents a non-specific antibody binding or low expression of a mutated Alox8 version is unclear.

A study in mice exposed to house dust mites revealed significantly higher 8-HETE present in the lung tissue [110]. Likewise, an increase in 9-HODE was detected in mice infected with the mold *Aspergillus fumigatus* [103]. Although Alox8 is the only enzyme, which can produce 8-HETE, 9-HODE may also be produced by other enzymes such as Alox15, cytochrome P450, or cyclooxygenases 1 and 2. Furthermore, a murine model of airway inflammation was reported to have increased Alox8 metabolites, indicating a role of Alox8 in lung inflammation [3].

Likewise, ALOX15B has been implicated in human airway inflammation. In the lung expression of ALOX15B is reported in type II pneumocytes and resident lung macrophages [31]. ALOX15B expression is elevated in the epithelial cells of severe versus mild asthmatic patients cultured in 3D airway models [32, 81]. Indeed, ALOX15B metabolite 15-HETE is elevated in the sputum of asthmatic patients [81], along with 13-HODE in asthmatic airways [69].

In contrast, cystic fibrosis showed decreased ALOX15B expression [86, 97], and chronic obstructive pulmonary disease reduced levels of 15-HETE [108]. Ringholz et al*.* [86] associated the decrease of ALOX15B with increased pro-inflammatory oxylipin leukotriene B4 and neutrophil infiltration in cystic fibrosis via transcellular ALOX5 activity. The authors propose a mechanism in which macrophage infiltration in lungs under inflammatory infections could dampen inflammation through production of pro-resolving LXA4 from either macrophage 8-HETE/15-HETE or neutrophil leukotriene A4, thus reducing the conversion to leukotriene B4 by neutrophils. One caveat is the lack of detection of tri-hydroxylated oxylipins via state-of-the-art mass spectrometry in vivo at a concentration thought to be high enough to elicit a biological response [90]. Ringholz et al*.* [86] detected LXA4 and leukotriene B4 via ELISA, which have been shown to be unreliable at detecting specialized pro-resolving lipid mediators [90]. These data may indicate an anti-inflammatory role of Alox8 and ALOX15B in lung diseases; however, further research directly linking oxylipins in the lungs of *Alox8*^−/−^ mice or airway inflammation models is needed.

### Alox8 and ALOX15B in the skin

The skin is a tissue which vastly differs in structural morphology between human and mouse. The human skin is approximately 100-µm thick, comprising 5–10 epidermal layers, whereas the mouse skin has only 2–3 epidermal layers of keratinocytes, resulting in a thickness of less than 25 µm [42]. Furthermore, the murine skin is absent of apocrine sweat glands, rete ridges, and arrector pili muscles, yet it contains an additional muscle layer, the panniculus carnosus, along with a synchronous hair cycle [54]. However, a commonality of ALOX15B and Alox8 is expression in the skin.

Alox8 was first discovered in murine skin upon phorbol ester stimulation [36], resulting in Alox8 expression in the terminal differentiated layers of the skin [47] and the production of 8-HETE. Transgenic mice with skin-specific Alox8 overexpression exhibited increased differentiation of the keratinocytes [74]. Furthermore, expression of Alox8 was detected in papillomas, yet expression is reduced with carcinoma [13]. Inducible expression of Alox8 in murine keratinocytes was shown to inhibit cell growth and proliferation, along with increased levels of cellular reactive oxygen species (ROS) [92]. Likewise, addition of 8-HETE was also shown to reduce the number of bromodeoxyuridine-positive cells, indicating reduced proliferation. Inhibition of p38 mitogen–activated protein kinase with SB203850 or ROS via the antioxidant *N*-acetyl-l-cysteine reversed effects of Alox8 overexpression [92]. Knockdown (KD) of ALOX15B in human lung adenocarcinoma cells revealed a reduction of cyclin A and D [120]. These results are consistent with the overexpression of human ALOX15B in murine keratinocytes [92]. Thus, as with ALOX15B, it might be assumed that Alox8 also affects the cell cycle through activation of p38 mitogen–activated protein kinase and cyclins.

Peroxisome proliferator–activated receptor (PPAR) signaling is associated with numerous cellular processes such as cell growth, differentiation, and tumorigenesis. Oxylipins produced by both human ALOX15B and murine Alox8 have been shown to act as ligands for PPAR isoforms [46, 118]. Activation of PPARα via 8-HETE in keratinocytes was shown to induce cell differentiation [74], whereas LOX inhibition with NDGA reduced keratin 1 expression [109].

Topical application of toll-like receptor 7/8 agonist imiquimod, a commonly used murine psoriasis model, was shown to induce the expression of Alox8 along with PPARδ [114]. Imiquimod treatment in combination with topical PPARδ inhibition via GSK3787 reduced epidermal proliferation, *Il17*, *Il23a*, *Il22*, *and Il-1b* expressions and IL-17-producing T cells. These data imply a role of Alox8 in murine psoriasis potentially signaling through PPARδ [44]. Likewise, upregulation of ALOX15B in lesional psoriatic in comparison to non-lesional skin was detected by in situ hybridization along with immunohistochemistry [78, 93]. These data are further backed up with many studies detecting elevated levels of 15-HETE and 13-HODE in psoriatic skin samples [14, 23, 24, 102, 107, 111] Contrastingly, reduced expression of *ALOX15B* [37] and 15-HETE levels in psoriatic skin [35, 44] have also been reported. Fogh et al. [25] performed intralesional injections of 15-HETE in patients with psoriasis vulgaris, revealing improved resolution, indicating an anti-inflammatory role of ALOX15B in psoriasis. Furthermore, spontaneous dermatitis found in NFκB inhibitor alpha (IκB-α)–deficient mice was associated with an increase in Alox8 expression [91]. These data indicate a role of Alox8 in the differentiation of keratinocytes and indicate that Alox8 plays a role in the resolution of skin inflammation.

### Alox8 and ALOX15B in cholesterol homeostasis and dyslipidemia

**Fig. 2:**
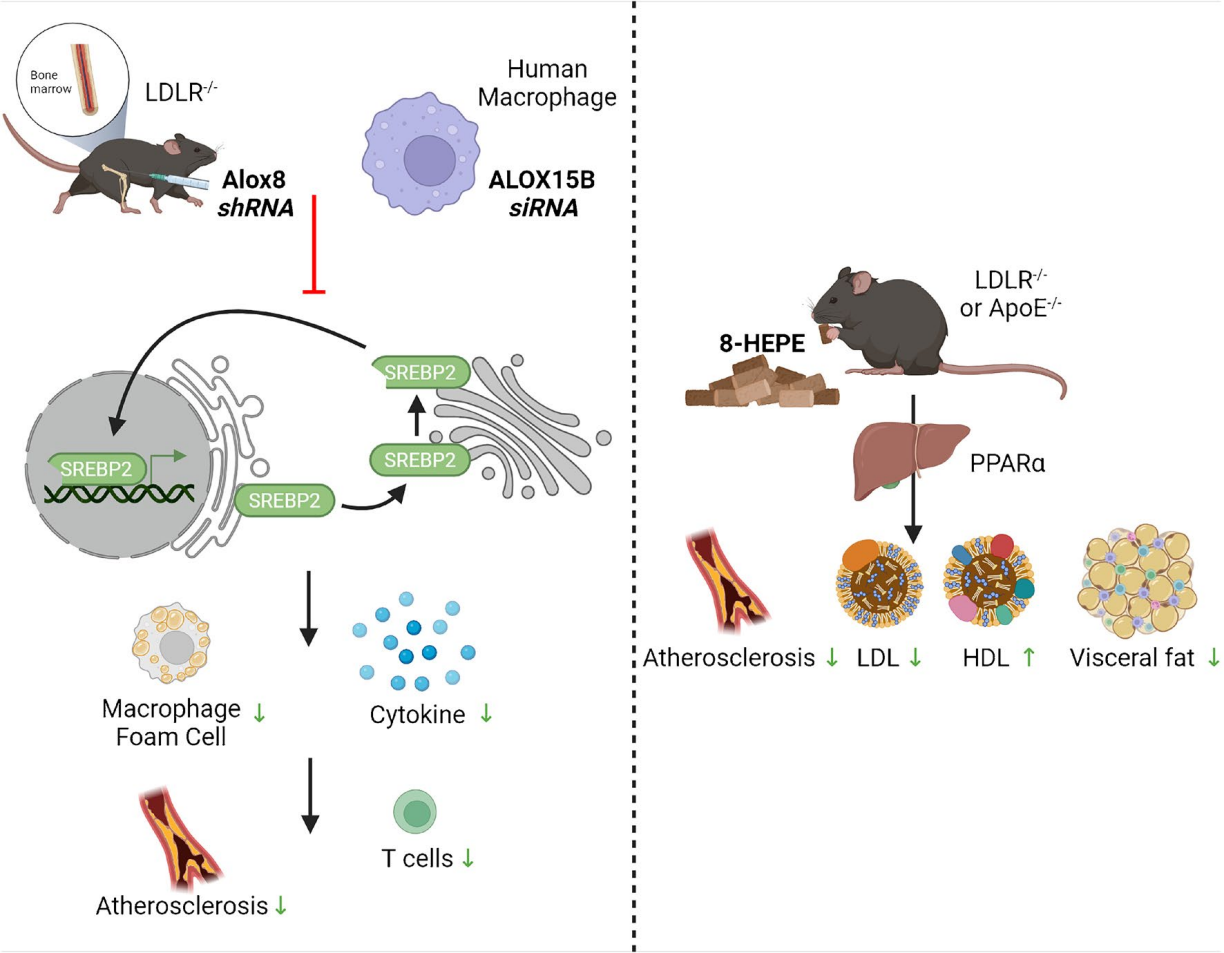
Contrasting roles of dietary 8-HEPE and modulation of Alox8/ALOX15B expression in the regulation of dyslipidemia. **a** *Ldlr*^−/−^ or *Apoe*^−/−^ mice fed with 8-HEPE supplementation to a Western diet exhibit a PPARα-dependent reduction in atherosclerosis, visceral fat, and circulating low-density lipoprotein (LDL) along with increased circulating high-density lipoprotein (HDL) levels. **b** Knockdown of *Alox8* or *ALOX15B* in macrophages show reduced sterol regulatory element-binding protein (SREBP) 2 signaling. Alterations to the major cholesterol biosynthesis regulator provokes reduced foam cell formation, cytokine secretion, atherosclerotic lesions, and T cell infiltration. AA, arachidonic acid; 8-HEPE, 8-hydroxy eicosapentaenoic acid

Both Alox8 [70] and ALOX15B [5, 101] have been implicated with cholesterol homeostasis in murine and human macrophages, respectively. Magnusson et al. [70] reported reduced foam cell formation with ALOX15B KD, which is in line with our findings of reduced sterol regulatory–binding protein (SREBP) 2 activity, the master transcriptional regulator of cholesterol biosynthesis [101], in primary human macrophages [5]. Furthermore, expression of Alox8 was reported in macrophages located in aortic atherosclerotic plaques of low-density lipoprotein knockout (*Ldlr*^−/−^) mice [70]. shRNA silencing of Alox8 via in vivo lentiviral transduction of bone marrow reduced atherosclerotic lesions, T cell infiltration, and IL-2 production. Moreover, addition of 8-HEPE to murine macrophage J774.1 cells resulted in significantly higher expression of cholesterol transporter ATP-binding cassette subfamily A member 1, fatty acid transporter Cd36, and IL-6 [87]. Collectively, these results indicate that Alox8 may play a role in dyslipidemia and cholesterol homeostasis (Fig. 2a), potentially through the production of 8-HEPE from EPA. However, EPA supplement alone was unable to elicit the same effects.

In addition to macrophage cholesterol homeostasis, treatment of mice with Alox8 metabolites was associated with altered cholesterol and lipid contents in the adipose tissue. *Ldlr*^−/−^ mice fed a Western diet with the addition of 8-HEPE showed a significant increase in body weight, along with reduced LDL and increased high-density lipoprotein (HDL) serum cholesterol levels [87] (Fig. 2b). Likewise, apolipoprotein E knockout (*Apoe*^−/−^) mice fed a Western diet with the addition of 8-HEPE had reduced blood glucose levels, along with a reduction in atherosclerotic lesion area [40] (Fig. 2b). 8-HEPE supplementation to mice fed on a high-fat diet revealed reduced visceral fat [40], in addition to the number of adipocytes in gonadal white adipose tissue [117]. In vitro experiment with murine pre-adipocyte 3T3-L1 cells revealed 8-HETE supplementation significantly reduced the number of lipid droplets [77], yet 8-HETE was shown to induce gene expression of adipocyte protein 2 [121]. Conversely, addition of 8-HEPE to murine pre-adipocyte cell line 3T3-F442A increased gene expression associated with fatty acid oxidation along with increased triglycerides and adipogenesis [118]. Cd44^+^ M2 macrophages derived from white adipose tissue had significantly higher 9-HODE levels than in CD44^−^ macrophages, and 9-HODE supplementation to platelet-derived growth factor receptor α–positive white adipose cells promoted the expression of genes associated with brown adipose tissue [64].

### Alox8/ALOX15B in inflammatory bowel disease

The correlation of ALOX15B and its metabolites in inflammatory bowel disease have been described. While ulcerative colitis [72] and Crohn’s disease [80] were associated with reduced ALOX15B expression and 15-HETE levels, raised 15-HETE levels were reported in inflamed colon tissue [123]. This elevation may indicate the role of ALOX15B during resolution of colon inflammation, which is supported by increased 15(*S*)-HETrE levels in colitis patients in remission [17]. Likewise, in a murine ulcerative colitis model, PD146176, a 15-lipoxygenase inhibitor, further reduced the body weight after dextran sulfate sodium treatment [72]. 8-HETE was increased in mice with induced intestinal ischemia. Following reperfusion, these levels returned to normal [30]. Although the function of Alox8 or its metabolites in the murine intestine has not been investigated, it could be postulated that the initial increase in 8-HETE is associated with an increase in the anti-inflammatory response to counteract the inflammatory damage from ischemia. In addition, mice exposed to ultrafine particles had increased levels of 9-HODE and a number of macrophages in the intestines [67]. Pochard et al. [80] demonstrated the role of 15-HETE in intestinal permeability barrier; expression of tight junction protein 1 was upregulated via AMP-activated protein kinase (AMPK) signaling. Following 15-HETE treatment in Caco-2 cells, increased transepithelial electrical resistance was also observed. Further research using *Alox8*^−/−^ or *Alox15b* knock-in mice in an inflammatory bowel model would help elucidate if higher levels of oxylipin products detected elevate intestinal inflammation.

### Alox8/ALOX15B in alcoholic liver disease

A murine model of alcoholic liver disease revealed increased levels of 9-HODE in both the liver and plasma. Furthermore, addition of 9-HODE to RAW264.7 murine monocyte-derived macrophages resulted in significant increases in the gene expression of tumor necrosis factor α, C-X-C motif chemokine ligand 2, and nitric oxide synthase 2 [115]. One study reported increases to both 15-LOX enzymes in human patients with alcoholic liver disease, along with 15-HETE as well as 9-/13-HODE levels [85]. These data indicate the potential role of ALOX15B/Alox8 in liver disease; however, further studies are needed to correlate the expression and activity of these enzymes in the liver.

### Alox8 in tumorigenesis

Like ALOX15B, Alox8 has also been shown to have an anti-tumorigenic role [56]. Overexpressing Alox8 mice, treated with phorbol esters to induce skin carcinogenesis, showed no differences in tumor incidence. However, tumor multiplicity was reduced. Additionally, a xenograft model with Alox8-overexpressing cells showed reduced tumor volume in vivo [56]. Inducible expression of Alox8 in pre-malignant murine keratinocytes inhibited cell proliferation, which was further exacerbated by stimulation with AA [92]. Specifically, AA-derived 8-HETE but not LA-derived 9-HODE was shown to inhibit cell growth [56, 92]. Conversely, shRNA-mediated KD of Alox8 in NIH-3T3 cells promoted tumorigenesis [68]. In addition, transplantation of hematopoietic stem and progenitor cells with shRNA-mediated Alox8 KD into mice resulted in a shorter lymphoma free survival than control mice [68]. KD of *Alox8* in primary lymphoma cells revealed elevated AA levels and a shunting of the AA metabolism to the cyclooxygenase pathway. This shunting was also present in cells with chromosome 11B deficiency (the homologous chromosome to human chromosome 17p where ALOX15B is located). In a B cell lymphoma model, KD of *Alox8* or *Alox8* in combination with cyclooxygenase 2 (*Ptgs2*) were injected into the tail vein of sublethally irradiated mice. Mice injected with *Alox8* shRNA tumors showed the poorest survival outcomes, which could be suppressed with simultaneous *Ptgs2* KD [82]. Cell viability of B cells, either with *Alox8* shRNA-mediated KD or chromosome 11B deficiency (11B3^+/−^), was reduced after inhibition of Ptgs2 in comparison to the control. The authors proposed that loss of ALOX15B or chromosome 17p deletions causes an accumulation of prostaglandin E2 and therefore inhibits apoptosis and B cell differentiation. Given that ALOX15B has been associated with ERK and AKT signaling pathways in human cancer cells [4], similar results may be postulated with Alox8 in murine carcinoma.

## Conclusion

Although located in the corresponding chromosomal location but given the differences in regiospecificity of ALOX15B and Alox8, divergent functionalities could be presumed. Nevertheless, similarities in the response to lung inflammation and cholesterol homeostasis in macrophages have been detected. Likewise, the involvement of Alox8 as a tumor suppressor corresponds to research found with some human tumors. However, there is a remarkable difference in prostate tissue: while ALOX15B is present in the normal human prostate, Alox8 is absent in the mouse prostate. Indeed, loss of ALOX15B is associated with prostate carcinoma, yet the presence of the murine homolog is not necessary for normal prostate function. Similarities in PPAR signaling can be expected, as other lipoxygenase-produced oxylipins can also activate PPAR isoforms. Future research should focus on the comparison of these two enzymes to see if murine models are appropriate to correlate with human ALOX15B activity.

## Data Availability

No datasets were generated or analyzed during the current study.

## References

[1] Adel S, Karst F, González-Lafont À et al (2016) Evolutionary alteration of ALOX15 specificity optimizes the biosynthesis of antiinflammatory and proresolving lipoxins. Proc Natl Acad Sci USA 113:E4266–E4275. 10.1073/pnas.1604029113 27412860 10.1073/pnas.1604029113 PMC4968705

[2] Alfardan R, Guo C, Toth LA et al. (2019) Impaired recovery from influenza A/X-31(H3N2) Infection in mice with 8-lipoxygenase deficiency. Med Sci (Basel) 7. 10.3390/medsci7040060 10.3390/medsci7040060 PMC6524062 31013822

[3] Bácsi A, Lucas R, Sütő MI et al (2022) An immune-shift induced by lycopene; from an eosinophil-dominant type towards an eosinophil/neutrophil-co-dominant type of airway inflammation. Food Funct 13:6534–6544. 10.1039/d2fo00875k 35642947 10.1039/d2fo00875k

[4] Benatzy Y, Palmer MA, Brüne B (2022) Arachidonate 15-lipoxygenase type B: regulation, function, and its role in pathophysiology. Front Pharmacol 13. 10.3389/fphar.2022.1042420 10.3389/fphar.2022.1042420 PMC9682198 36438817

[5] Benatzy Y, Palmer MA, Lütjohann D, Ohno RI, Kampschulte N, Schebb NH, Fuhrmann DC, Snodgrass RG, Brüne B (2024) ALOX15B controls macrophage cholesterol homeostasis via lipid peroxidation, ERK1/2 and SREBP2. Redox Biol 72:103149. 10.1016/j.redox.2024.103149 10.1016/j.redox.2024.103149 PMC11002893 38581859

[6] Bender G, Schexnaydre EE, Murphy RC et al (2016) Membrane-dependent activities of human 15-LOX-2 and its murine counterpart: implications for murine models of atherosclerosis. J Biol Chem 291:19413–19424. 10.1074/jbc.M116.741454 27435673 10.1074/jbc.M116.741454 PMC5016680

[7] Borngräber S, Browner M, Gillmor S et al (1999) Shape and specificity in mammalian 15-lipoxygenase active site. The functional interplay of sequence determinants for the reaction specificity. J Biol Chem 274:37345–37350. 10.1074/jbc.274.52.37345 10601303 10.1074/jbc.274.52.37345

[8] Borngräber S, Kuban RJ, Anton M et al (1996) Phenylalanine 353 is a primary determinant for the positional specificity of mammalian 15-lipoxygenases. J Mol Biol 264:1145–1153. 10.1006/jmbi.1996.0702 9000636 10.1006/jmbi.1996.0702

[9] Bouchaoui H, Mahoney-Sanchez L, Garçon G et al (2023) ACSL4 and the lipoxygenases 15/15B are pivotal for ferroptosis induced by iron and PUFA dyshomeostasis in dopaminergic neurons. Free Radic Biol Med 195:145–157. 10.1016/j.freeradbiomed.2022.12.086 36581060 10.1016/j.freeradbiomed.2022.12.086

[10] Brash AR, Boeglin WE, Chang MS et al (1996) Purification and molecular cloning of an 8R-lipoxygenase from the coral Plexaura homomalla reveal the related primary structures of R- and S-lipoxygenases. J Biol Chem 271:20949–20957. 10.1074/jbc.271.34.20949 8702854 10.1074/jbc.271.34.20949

[11] Brash AR, Boeglin WE, Chang MS (1997) Discovery of a second 15S-lipoxygenase in humans. Proc Natl Acad Sci USA 94:6148–6152. 10.1073/pnas.94.12.6148 9177185 10.1073/pnas.94.12.6148 PMC21017

[12] Brash AR, Jisaka M, Boeglin WE et al (1999) Molecular cloning of a second human 15S-lipoxygenase and its murine homologue, an 8S-lipoxygenase. Their relationship to other mammalian lipoxygenases. Adv Exp Med Biol 447:29–36. 10.1007/978-1-4615-4861-4_3 10086180 10.1007/978-1-4615-4861-4_3

[13] Bürger F, Krieg P, Kinzig A et al (1999) Constitutive expression of 8-lipoxygenase in papillomas and clastogenic effects of lipoxygenase-derived arachidonic acid metabolites in keratinocytes. Mol Carcinog 24:108–117. 10.1002/(sici)1098-2744(199902)24:2%3c108:aid-mc5%3e3.0.co;2-r 10078938 10.1002/(sici)1098-2744(199902)24:2<108::aid-mc5>3.0.co;2-r

[14] Camp R, Mallet AI, Woollard PM et al (1983) The identification of hydroxy fatty acids in psoriatic skin. Prostaglandins 26:431–447. 10.1016/0090-6980(83)90178-8 6419288 10.1016/0090-6980(83)90178-8

[15] Coffa G, Brash AR (2004) A single active site residue directs oxygenation stereospecificity in lipoxygenases: stereocontrol is linked to the position of oxygenation. Proc Natl Acad Sci USA 101:15579–15584. 10.1073/pnas.0406727101 15496467 10.1073/pnas.0406727101 PMC524819

[16] Daurkin I, Eruslanov E, Stoffs T et al (2011) Tumor-associated macrophages mediate immunosuppression in the renal cancer microenvironment by activating the 15-lipoxygenase-2 pathway. Cancer Res 71:6400–6409. 10.1158/0008-5472.CAN-11-1261 21900394 10.1158/0008-5472.CAN-11-1261

[17] Diab J, Al-Mahdi R, Gouveia-Figueira S et al (2019) A quantitative analysis of colonic mucosal oxylipins and endocannabinoids in treatment-naïve and deep remission ulcerative colitis patients and the potential link with cytokine gene expression. Inflamm Bowel Dis 25:490–497. 10.1093/ibd/izy349 30476077 10.1093/ibd/izy349 PMC6383859

[18] Drake TA, Schadt E, Hannani K et al (2001) Genetic loci determining bone density in mice with diet-induced atherosclerosis. Physiol Genom 5:205–215. 10.1152/physiolgenomics.2001.5.4.205 10.1152/physiolgenomics.2001.5.4.205 11328966

[19] Ebert R, Cumbana R, Lehmann C et al (2020) Long-term stimulation of toll-like receptor-2 and -4 upregulates 5-LO and 15-LO-2 expression thereby inducing a lipid mediator shift in human monocyte-derived macrophages. Biochim Biophys Acta Mol Cell Biol Lipids 1865:158702. 10.1016/j.bbalip.2020.158702 32222425 10.1016/j.bbalip.2020.158702

[20] Fischer SM, Baldwin JK, Jasheway DW et al (1988) Phorbol ester induction of 8-lipoxygenase in inbred SENCAR (SSIN) but not C57BL/6J mice correlated with hyperplasia, edema, and oxidant generation but not ornithine decarboxylase induction. Cancer Res 48:658–664 3335028

[21] Fischer SM, Fürstenberger G, Marks F et al (1987) Events associated with mouse skin tumor promotion with respect to arachidonic acid metabolism: a comparison between SENCAR and NMRI mice. Cancer Res 47:3174–3179 3107806

[22] Flohé L, Toppo S, Orian L (2022) The glutathione peroxidase family: discoveries and mechanism. Free Radic Biol Med 187:113–122. 10.1016/j.freeradbiomed.2022.05.003 35580774 10.1016/j.freeradbiomed.2022.05.003

[23] Fogh K, Herlin T, Kragballe K (1989) Eicosanoids in skin of patients with atopic dermatitis: prostaglandin E and leukotriene B are present in biologically active concentrations. J Allergy Clin Immunol 83:450–455. 10.1016/0091-6749(89)90132-2 2537352 10.1016/0091-6749(89)90132-2

[24] Fogh K, Kiil J, Herlin T et al (1987) Heterogeneous distribution of lipoxygenase products in psoriatic skin lesions. Arch Dermatol Res 279:504–511. 10.1007/BF00413280 2829753 10.1007/BF00413280

[25] Fogh K, Søgaard H, Herlin T et al (1988) Improvement of psoriasis vulgaris after intralesional injections of 15-hydroxyeicosatetraenoic acid (15-HETE). J Am Acad Dermatol 18:279–285. 10.1016/S0190-9622(88)70040-7 3346412 10.1016/s0190-9622(88)70040-7

[26] Freire-Moar J, Alavi-Nassab A, Ng M et al (1995) Cloning and characterization of a murine macrophage lipoxygenase. Biochim Biophys Acta 1254:112–116. 10.1016/0005-2760(94)00199-9 7811740 10.1016/0005-2760(94)00199-9

[27] Funk CD, Chen X-S, Johnson EN et al (2002) Lipoxygenase genes and their targeted disruption. Prostaglandins Other Lipid Mediat 68–69:303–312. 10.1016/s0090-6980(02)00036-9 12432925 10.1016/s0090-6980(02)00036-9

[28] Fürstenberger G, Hagedorn H, Jacobi T et al (1991) Characterization of an 8-lipoxygenase activity induced by the phorbol ester tumor promoter 12-O-tetradecanoylphorbol-13-acetate in mouse skin in vivo. J Biol Chem 266:15738–15745. 10.1016/S0021-9258(18)98471-1 1874732

[29] Fürstenberger G, Marks F, Krieg P (2002) Arachidonate 8(S)-lipoxygenase. Prostaglandins Other Lipid Mediat 68–69:235–243. 10.1016/S0090-6980(02)00033-3 12432921 10.1016/s0090-6980(02)00033-3

[30] Gobbetti T, Le Faouder P, Bertrand J et al (2013) Polyunsaturated fatty acid metabolism signature in ischemia differs from reperfusion in mouse intestine. PLoS ONE 8:e75581. 10.1371/journal.pone.0075581 24073272 10.1371/journal.pone.0075581 PMC3779198

[31] Gonzalez AL, Roberts RL, Massion PP et al (2004) 15-Lipoxygenase-2 expression in benign and neoplastic lung: an immunohistochemical study and correlation with tumor grade and proliferation. Hum Pathol 35:840–849. 10.1016/j.humpath.2004.04.001 15257547 10.1016/j.humpath.2004.04.001

[32] Gras D, Bourdin A, Vachier I et al (2012) An ex vivo model of severe asthma using reconstituted human bronchial epithelium. J Allergy Clin Immunol 129:1259-1266.e1. 10.1016/j.jaci.2012.01.073 22409990 10.1016/j.jaci.2012.01.073

[33] Green AR, Barbour S, Horn T et al (2016) Strict regiospecificity of human epithelial 15-lipoxygenase-2 delineates its transcellular synthesis potential. Biochemistry 55:2832–2840. 10.1021/acs.biochem.5b01339 27145229 10.1021/acs.biochem.5b01339 PMC5657383

[34] Green AR, Freedman C, Tena J et al (2018) 5 S,15 S-Dihydroperoxyeicosatetraenoic Acid (5,15-diHpETE) as a lipoxin intermediate: reactivity and kinetics with human leukocyte 5-lipoxygenase, platelet 12-lipoxygenase, and reticulocyte 15-lipoxygenase-1. Biochemistry 57:6726–6734. 10.1021/acs.biochem.8b00889 30407793 10.1021/acs.biochem.8b00889 PMC7270142

[35] Grøn B, Iversen L, Ziboh V et al (1993) Monohydroxy fatty acids esterified to phospholipids are decreased in lesional psoriatic skin. Arch Dermatol Res 285:449–454. 10.1007/BF00376816 8274032 10.1007/BF00376816

[36] Gschwendt M, Fürstenberger G, Kittstein W et al (1986) Generation of the arachidonic acid metabolite 8-HETE by extracts of mouse skin treated with phorbol ester in vivo; identification by 1H-n.m.r. and GC-MS spectroscopy. Carcinogenesis 7:449–455. 10.1093/carcin/7.3.449 3081276 10.1093/carcin/7.3.449

[37] Gudjonsson JE, Ding J, Li X et al (2009) Global gene expression analysis reveals evidence for decreased lipid biosynthesis and increased innate immunity in uninvolved psoriatic skin. J Invest Dermatol 129:2795–2804. 10.1038/jid.2009.173 19571819 10.1038/jid.2009.173 PMC2783967

[38] Hamberg M, Samuelsson B (1980) Stereochemistry in the formation of 9-hydroxy-10,12-octadecadienoic acid and 13-hydroxy-9,11-octadecadienoic acid from linoleic acid by fatty acid cyclooxygenase. Biochim Biophys Acta 617:545–547. 10.1016/0005-2760(80)90022-3 6768399 10.1016/0005-2760(80)90022-3

[39] Heidt M, Fürstenberger G, Vogel S et al (2000) Diversity of mouse lipoxygenases: identification of a subfamily of epidermal isozymes exhibiting a differentiation-dependent mRNA expression pattern. Lipids 35:701–707. 10.1007/s11745-000-0576-0 10941870 10.1007/s11745-000-0576-0

[40] Hirose M, Ibi M, Saito M et al (2019) Abstract 11153: 8-Hydroxyeicosapentaenoic acid concentrated materials from pacific krill suppresses atherosclerosis in apolipoprotein E-deficient mice. Circulation 140:A11153–A11153. 10.1161/circ.140.suppl_1.11153

[41] Hughes MA, Brash AR (1991) Investigation of the mechanism of biosynthesis of 8-hydroxyeicosatetraenoic acid in mouse skin. Biochim Biophys Acta 1081:347–354. 10.1016/0005-2760(91)90292-P 1900207 10.1016/0005-2760(91)90292-p

[42] Hultén LM, Olson FJ, Åberg H et al (2010) 15-Lipoxygenase-2 is expressed in macrophages in human carotid plaques and regulated by hypoxia-inducible factor-1α. Eur J Clin Invest 40:11–17. 10.1111/j.1365-2362.2009.02223.x 19912316 10.1111/j.1365-2362.2009.02223.x

[43] Ivanov I, Heydeck D, Hofheinz K et al (2010) Molecular enzymology of lipoxygenases. Arch Biochem Biophys 503:161–174. 10.1016/j.abb.2010.08.016 20801095 10.1016/j.abb.2010.08.016

[44] Iversen L, Kragballe K (2000) Arachidonic acid metabolism in skin health and disease. Prostaglandins Other Lipid Mediat 63:25–42. 10.1016/s0090-6980(00)00095-2 11104339 10.1016/s0090-6980(00)00095-2

[45] Jansen C, Hofheinz K, Vogel R et al (2011) Stereocontrol of arachidonic acid oxygenation by vertebrate lipoxygenases: newly cloned zebrafish lipoxygenase 1 does not follow the Ala-versus-Gly concept. J Biol Chem 286:37804–37812. 10.1074/jbc.M111.259242 21880725 10.1074/jbc.M111.259242 PMC3199522

[46] Jisaka M, Iwanaga C, Takahashi N et al (2005) Double dioxygenation by mouse 8S-lipoxygenase: specific formation of a potent peroxisome proliferator-activated receptor α agonist. Biochem Biophys Res Commun 338:136–143. 10.1016/j.bbrc.2005.08.029 16112079 10.1016/j.bbrc.2005.08.029

[47] Jisaka M, Kim RB, Boeglin WE et al (1997) Molecular cloning and functional expression of a phorbol ester-inducible 8S-lipoxygenase from mouse skin. J Biol Chem 272:24410–24416. 10.1074/jbc.272.39.24410 9305900 10.1074/jbc.272.39.24410

[48] Jisaka M, Kim RB, Boeglin WE et al (2000) Identification of amino acid determinants of the positional specificity of mouse 8S-lipoxygenase and human 15S-lipoxygenase-2. J Biol Chem 275:1287–1293. 10.1074/jbc.275.2.1287 10625675 10.1074/jbc.275.2.1287

[49] Kahnt AS, Schebb NH, Steinhilber D (2023) Formation of lipoxins and resolvins in human leukocytes. Prostaglandins Other Lipid Mediat 166:106726. 10.1016/j.prostaglandins.2023.106726 36878381 10.1016/j.prostaglandins.2023.106726

[50] Kakularam KR, Canyelles-Niño M, Chen X et al. (2023) Functional characterization of mouse and human arachidonic acid lipoxygenase 15B (ALOX15B) orthologs and of their mutants exhibiting humanized and murinized reaction specificities. Int J Mol Sci 24. 10.3390/ijms241210046 10.3390/ijms241210046 PMC10298594 37373195

[51] Kapralov AA, Yang Q, Dar HH et al (2020) Redox lipid reprogramming commands susceptibility of macrophages and microglia to ferroptotic death. Nat Chem Biol 16:278–290. 10.1038/s41589-019-0462-8 32080625 10.1038/s41589-019-0462-8 PMC7233108

[52] Kawajiri H, Hsi LC, Kamitani H et al (2002) Arachidonic and linoleic acid metabolism in mouse intestinal tissue: evidence for novel lipoxygenase activity. Arch Biochem Biophys 398:51–60. 10.1006/abbi.2001.2685 11811948 10.1006/abbi.2001.2685

[53] Kawajiri H, Piao Y, Takahashi Y et al (2005) Synthesis of 8,9-leukotriene A4 by murine 8-lipoxygenase. Biochem Biophys Res Commun 338:144–148. 10.1016/j.bbrc.2005.08.099 16143298 10.1016/j.bbrc.2005.08.099

[54] Kensler KH, Regan MM, Heng YJ et al (2019) Prognostic and predictive value of androgen receptor expression in postmenopausal women with estrogen receptor-positive breast cancer: results from the Breast International Group Trial 1–98. Breast Cancer Res 21:30. 10.1186/s13058-019-1118-z 30795773 10.1186/s13058-019-1118-z PMC6387478

[55] Kilty I, Logan A, Vickers PJ (1999) Differential characteristics of human 15-lipoxygenase isozymes and a novel splice variant of 15S-lipoxygenase. Eur J Biochem 266:83–93. 10.1046/j.1432-1327.1999.00818.x 10542053 10.1046/j.1432-1327.1999.00818.x

[56] Kim E, Rundhaug JE, Benavides F et al (2005) An antitumorigenic role for murine 8S-lipoxygenase in skin carcinogenesis. Oncogene 24:1174–1187. 10.1038/sj.onc.1208269 15558016 10.1038/sj.onc.1208269

[57] Kobe MJ, Neau DB, Mitchell CE et al (2014) The structure of human 15-lipoxygenase-2 with a substrate mimic. J Biol Chem 289:8562–8569. 10.1074/jbc.M113.543777 24497644 10.1074/jbc.M113.543777 PMC3961679

[58] Krieg P, Kinzig A, Heidt M et al (1998) cDNA cloning of a 8-lipoxygenase and a novel epidermis-type lipoxygenase from phorbol ester-treated mouse skin. Biochim Biophys Acta 1391:7–12. 10.1016/s0005-2760(97)00214-2 9518531 10.1016/s0005-2760(97)00214-2

[59] Kühn H, Barnett J, Grunberger D et al (1993) Overexpression, purification and characterization of human recombinant 15-lipoxygenase. Biochim Biophys Acta 1169:80–89. 10.1016/0005-2760(93)90085-N 8334154 10.1016/0005-2760(93)90085-n

[60] Kuhn H, Humeniuk L, Kozlov N et al (2018) The evolutionary hypothesis of reaction specificity of mammalian ALOX15 orthologs. Prog Lipid Res 72:55–74. 10.1016/j.plipres.2018.09.002 30237084 10.1016/j.plipres.2018.09.002

[61] Kühn H, Schewe T, Rapoport SM (1986) The stereochemistry of the reactions of lipoxygenases and their metabolites. Proposed nomenclature of lipoxygenases and related enzymes. Adv Enzymol Relat Areas Mol Biol 58:273–311. 10.1002/9780470123041.ch7 3087142 10.1002/9780470123041.ch7

[62] Kühn H, Wiesner R, Schewe T et al (1983) Reticulocyte lipoxygenase exhibits both n-6 and n-9 activities. FEBS Lett 153:353–356. 10.1016/0014-5793(83)80641-3 6413249 10.1016/0014-5793(83)80641-3

[63] Kutzner L, Goloshchapova K, Heydeck D et al (2017) Mammalian ALOX15 orthologs exhibit pronounced dual positional specificity with docosahexaenoic acid. Biochim Biophys Acta Mol Cell Biol Lipids 1862:666–675. 10.1016/j.bbalip.2017.04.001 28400162 10.1016/j.bbalip.2017.04.001

[64] Lee Y-H, Kim S-N, Kwon H-J et al (2015) Adipogenic role of alternatively activated macrophages in β-adrenergic remodeling of white adipose tissue. Am J Physiol Regul Integr Comp Physiol 310:R55-65. 10.1152/ajpregu.00355.2015 26538237 10.1152/ajpregu.00355.2015 PMC4796635

[65] Lehmann C, Homann J, Ball A-K et al (2015) Lipoxin and resolvin biosynthesis is dependent on 5-lipoxygenase activating protein. FASEB J 29:5029–5043. 10.1096/fj.15-275487 26289316 10.1096/fj.15-275487

[66] Lehmann WD, Stephan M, Fürstenberger G (1992) Profiling assay for lipoxygenase products of linoleic and arachidonic acid by gas chromatography-mass spectrometry. Anal Biochem 204:158–170. 10.1016/0003-2697(92)90156-2 1514683 10.1016/0003-2697(92)90156-2

[67] Li R, Navab K, Hough G et al (2014) Effect of exposure to atmospheric ultrafine particles on production of free fatty acids and lipid metabolites in the mouse small intestine. Environ Health Perspect 123:34–41. 10.1289/ehp.1307036 25170928 10.1289/ehp.1307036 PMC4286268

[68] Liu Y, Chen C, Xu Z et al (2016) Deletions linked to TP53 loss drive cancer through p53-independent mechanisms. Nature 531:471–475. 10.1038/nature17157 26982726 10.1038/nature17157 PMC4836395

[69] Mabalirajan U, Rehman R, Ahmad T et al (2013) Linoleic acid metabolite drives severe asthma by causing airway epithelial injury. Sci Rep 3:1349. 10.1038/srep01349 23443229 10.1038/srep01349 PMC3583002

[70] Magnusson LU, Lundqvist A, Karlsson MN et al (2012) Arachidonate 15-lipoxygenase type B knockdown leads to reduced lipid accumulation and inflammation in atherosclerosis. PLoS ONE 7:e43142. 10.1371/journal.pone.0043142 22912809 10.1371/journal.pone.0043142 PMC3422220

[71] Maiorino M, Thomas JP, Girotti AW et al (1991) Reactivity of phospholipid hydroperoxide glutathione peroxidase with membrane and lipoprotein lipid hydroperoxides. Free Radic Res Commun 12–13(Pt 1):131–135. 10.3109/10715769109145777 10.3109/10715769109145777 2071029

[72] Mangino MJ, Brounts L, Harms B et al (2006) Lipoxin biosynthesis in inflammatory bowel disease. Prostaglandins Other Lipid Mediat 79:84–92. 10.1016/j.prostaglandins.2005.10.004 16516812 10.1016/j.prostaglandins.2005.10.004

[73] Mehrabian M, Allayee H, Stockton J et al (2005) Integrating genotypic and expression data in a segregating mouse population to identify 5-lipoxygenase as a susceptibility gene for obesity and bone traits. Nat Genet 37:1224–1233. 10.1038/ng1619 16200066 10.1038/ng1619

[74] Muga S, Thuillier P, Pavone A et al (2000) 8s-Lipoxygenase products activate peroxisome proliferator-activated receptor ?? and induce differentiation in murine keratinocytes. Cell Growth Differ 11:447–454 10965849

[75] Neau DB, Bender G, Boeglin WE et al (2014) Crystal structure of a lipoxygenase in complex with substrate: the arachidonic acid-binding site of 8R-lipoxygenase. J Biol Chem 289:31905–31913. 10.1074/jbc.M114.599662 25231982 10.1074/jbc.M114.599662 PMC4231669

[76] Newcomer ME, Brash AR (2015) The structural basis for specificity in lipoxygenase catalysis. Protein Sci 24:298–309. 10.1002/pro.2626 25524168 10.1002/pro.2626 PMC4353356

[77] Pal A, Sun S, Armstrong M et al (2021) Beneficial effects of eicosapentaenoic acid on the metabolic profile of obese female mice entails upregulation of HEPEs and increased abundance of enteric Akkermansia Muciniphila. Biochim Biophys Acta Mol Cell Biol Lipids 1867:159059. 10.1016/j.bbalip.2021.159059 34619367 10.1016/j.bbalip.2021.159059 PMC8627244

[78] Palmer M, Bürger C, Benatzy Y et al (2023) 177 ALOX15B knockdown augments keratinocyte inflammation. J Investig Dermatol 143:S362. 10.1016/j.jid.2023.09.185

[79] Perry SC, Horn T, Tourdot BE et al (2020) Role of Human 15-lipoxygenase-2 in the biosynthesis of the lipoxin intermediate, 5S,15S-diHpETE, implicated with the altered positional specificity of human 15-lipoxygenase-1. Biochemistry 59:4118–4130. 10.1021/acs.biochem.0c00622 33048542 10.1021/acs.biochem.0c00622 PMC7721368

[80] Pochard C, Coquenlorge S, Jaulin J et al (2016) Defects in 15-HETE production and control of epithelial permeability by human enteric glial cells from patients with Crohn’s disease. Gastroenterology 150:168–180. 10.1053/j.gastro.2015.09.038 26433161 10.1053/j.gastro.2015.09.038

[81] Profita M, Sala A, Riccobono L et al (2000) 15-Lipoxygenase expression and 15(S)-hydroxyeicoisatetraenoic acid release and reincorporation in induced sputum of asthmatic subjects. J Allergy Clin Immunol 105:711–716. 10.1067/mai.2000.105122 10756220 10.1067/mai.2000.105122

[82] Qi L, Pan X, Chen X et al (2023) COX-2/PGE2 upregulation contributes to the chromosome 17p-deleted lymphoma. Oncogenesis 12:5. 10.1038/s41389-023-00451-9 36750552 10.1038/s41389-023-00451-9 PMC9905509

[83] Qiao N, Takahashi Y, Takamatsu H et al (1999) Leukotriene A synthase activity of purified mouse skin arachidonate 8-lipoxygenase expressed in Escherichia coli. Biochim Biophys Acta 1438:131–139. 10.1016/s1388-1981(99)00035-9 10216287 10.1016/s1388-1981(99)00035-9

[84] Rademacher M, Kuhn H, Borchert A (2020) Systemic deficiency of mouse arachidonate 15-lipoxygenase induces defective erythropoiesis and transgenic expression of the human enzyme rescues this phenotype. FASEB J 34:14318–14335. 10.1096/fj.202000408RR 32918502 10.1096/fj.202000408RR

[85] Raszeja-Wyszomirska J, Safranow K, Milkiewicz M et al (2012) Lipidic last breath of life in patients with alcoholic liver disease. Prostaglandins Other Lipid Mediat 99:51–56. 10.1016/j.prostaglandins.2012.06.001 22706383 10.1016/j.prostaglandins.2012.06.001

[86] Ringholz FC, Buchanan PJ, Clarke DT et al (2014) Reduced 15-lipoxygenase 2 and lipoxin A4/leukotriene B4 ratio in children with cystic fibrosis. Eur Respir J 44:394–404. 10.1183/09031936.00106013 24696116 10.1183/09031936.00106013

[87] Saito M, Ishida N, Yamada H et al (2020) 8-HEPE-concentrated materials from pacific krill improve plasma cholesterol levels and hepatic steatosis in high cholesterol diet-fed low-density lipoprotein (LDL) receptor-deficient mice. Biol Pharm Bull 43:919–924. 10.1248/bpb.b20-00162 32475913 10.1248/bpb.b20-00162

[88] Schäfer M, Kakularam KR, Reisch F et al. (2022) Male knock-in mice expressing an arachidonic acid lipoxygenase 15B (Alox15B) with humanized reaction specificity are prematurely growth arrested when aging. Biomedicines 10. 10.3390/biomedicines10061379 10.3390/biomedicines10061379 PMC9220125 35740398

[89] Schäfer M, Reisch F, Labuz D et al. (2023) Humanization of the reaction specificity of mouse Alox15b inversely modified the susceptibility of corresponding knock-in mice in two different animal inflammation models. Int J Mol Sci 24. 10.3390/ijms241311034 10.3390/ijms241311034 PMC10341735 37446212

[90] Schebb NH, Kühn H, Kahnt AS et al (2022) Formation, signaling and occurrence of specialized pro-resolving lipid mediators-what is the evidence so far? Front Pharmacol 13:838782. 10.3389/fphar.2022.838782 35308198 10.3389/fphar.2022.838782 PMC8924552

[91] Schneider C, David Strayhorn W, Brantley DM et al (2004) Upregulation of 8-lipoxygenase in the dermatitis of IκB-α-deficient mice. J Investig Dermatol 122:691–698. 10.1111/j.0022-202X.2004.22329.x 15086555 10.1111/j.0022-202X.2004.22329.x

[92] Schweiger D, Fürstenberger G, Krieg P (2007) Inducible expression of 15-lipoxygenase-2 and 8-lipoxygenase inhibits cell growth via common signaling pathways. J Lipid Res 48:553–564. 10.1194/jlr.M600311-JLR200 17164225 10.1194/jlr.M600311-JLR200

[93] Setsu N, Matsuura H, Hirakawa S et al (2006) Interferon-gamma-induced 15-lipoxygenase-2 expression in normal human epidermal keratinocytes and a pathogenic link to psoriasis vulgaris. Eur J Dermatol 16:141–145 16581564

[94] Shi H, Carion TW, Jiang Y et al (2017) A regulatory role for β-adrenergic receptors regarding the resolvin D1 (RvD1) pathway in the diabetic retina. PLoS ONE 12:e0185383. 10.1371/journal.pone.0185383 29095817 10.1371/journal.pone.0185383 PMC5667888

[95] Shimizu T, Izumi T, Ohishi N et al (1987) Biosynthesis and further transformations of leukotriene A4. Adv Prostaglandin Thromboxane Leukot Res 17A:64–68 2821769

[96] Shin K-C, Kang W-R, Seo M-J et al (2019) Production of 8S- and 10S-hydroxy polyunsaturated fatty acids by recombinant Escherichia coli cells expressing mouse arachidonate 8S-lipoxygenase. Biotechnol Lett 41:575–582. 10.1007/s10529-019-02659-5 30825045 10.1007/s10529-019-02659-5

[97] Shum M, London CM, Briottet M et al (2022) CF patients’ airway epithelium and sex contribute to biosynthesis defects of pro-resolving lipids. Front Immunol 13:915261. 10.3389/fimmu.2022.915261 35784330 10.3389/fimmu.2022.915261 PMC9244846

[98] Sigal E, Craik CS, Highland E et al (1988) Molecular cloning and primary structure of human 15-lipoxygenase. Biochem Biophys Res Commun 157:457–464. 10.1016/s0006-291x(88)80271-7 3202857 10.1016/s0006-291x(88)80271-7

[99] Sigal E, Grunberger D, Craik CS et al (1988) Arachidonate 15-lipoxygenase (omega-6 lipoxygenase) from human leukocytes. Purification and structural homology to other mammalian lipoxygenases. J Biol Chem 263:5328–5332 3356688

[100] Sloane DL, Leung R, Craik CS et al (1991) A primary determinant for lipoxygenase positional specificity. Nature 354:149–152. 10.1038/354149a0 1944593 10.1038/354149a0

[101] Snodgrass RG, Zezina E, Namgaladze D et al (2018) A novel function for 15-lipoxygenases in cholesterol homeostasis and CCL17 production in human macrophages. Front Immunol 9:1906. 10.3389/fimmu.2018.01906 30197642 10.3389/fimmu.2018.01906 PMC6117383

[102] Sorokin AV, Domenichiello AF, Dey AK et al (2018) Bioactive lipid mediator profiles in human psoriasis skin and blood. J Invest Dermatol 138:1518–1528. 10.1016/j.jid.2018.02.003 29454560 10.1016/j.jid.2018.02.003 PMC6121727

[103] Steffan BN, Calise D, Park SC et al. (2023) Loss of the mammalian G-protein coupled receptor, G2A, modulates severity of invasive pulmonary aspergillosis. Front Immunol 14. 10.3389/fimmu.2023.1173544 10.3389/fimmu.2023.1173544 PMC10331294 37435068

[104] Suardíaz R, Jambrina PG, Masgrau L et al (2016) Understanding the mechanism of the hydrogen abstraction from arachidonic acid catalyzed by the human enzyme 15-lipoxygenase-2. A quantum mechanics/molecular mechanics free energy simulation. J Chem Theory Comput 12:2079–2090. 10.1021/acs.jctc.5b01236 26918937 10.1021/acs.jctc.5b01236

[105] Subbarayan V, Xu X-C, Kim J et al (2005) Inverse relationship between 15-lipoxygenase-2 and PPAR-gamma gene expression in normal epithelia compared with tumor epithelia. Neoplasia 7:280–293. 10.1593/neo.04457 15799828 10.1593/neo.04457 PMC1501140

[106] Suraneni MV, Schneider-Broussard R, Moore JR et al (2010) Transgenic expression of 15-lipoxygenase 2 (15-LOX2) in mouse prostate leads to hyperplasia and cell senescence. Oncogene 29:4261–4275. 10.1038/onc.2010.197 20514017 10.1038/onc.2010.197 PMC3042242

[107] Takeichi T, Kinoshita F, Tanaka H et al (2019) The lipoxygenase-hepoxilin pathway is activated in cutaneous plaque lesions of psoriasis. J Cutan Immunol Allergy 2:15–24. 10.1002/cia2.12039

[108] Teopompi E, Risé P, Pisi R et al (2019) Arachidonic acid and docosahexaenoic acid metabolites in the airways of adults with cystic fibrosis: effect of docosahexaenoic acid supplementation. Front Pharmacol 10:938. 10.3389/fphar.2019.00938 31507425 10.3389/fphar.2019.00938 PMC6716427

[109] Thuillier P, Brash AR, Kehrer JP et al (2002) Inhibition of peroxisome proliferator-activated receptor (PPAR)-mediated keratinocyte differentiation by lipoxygenase inhibitors. Biochem J 366:901–910. 10.1042/BJ20020377 12069687 10.1042/BJ20020377 PMC1222830

[110] Turi KN, Michel CR, Manke J et al. (2023) Multi-omics analysis of lung tissue demonstrates changes to lipid metabolism during allergic sensitization in mice. Metabolites 13. 10.3390/metabo13030406 10.3390/metabo13030406 PMC10054742 36984845

[111] Tyrrell VJ, Ali F, Boeglin WE et al (2021) Lipidomic and transcriptional analysis of the linoleoyl-omega-hydroxyceramide biosynthetic pathway in human psoriatic lesions. J Lipid Res 62:100094. 10.1016/j.jlr.2021.100094 34171322 10.1016/j.jlr.2021.100094 PMC8326207

[112] Vogel R, Jansen C, Roffeis J et al (2010) Applicability of the triad concept for the positional specificity of mammalian lipoxygenases. J Biol Chem 285:5369–5376. 10.1074/jbc.M109.057802 20026599 10.1074/jbc.M109.057802 PMC2820765

[113] Walther M, Roffeis J, Jansen C et al (2009) Structural basis for pH-dependent alterations of reaction specificity of vertebrate lipoxygenase isoforms. Biochim Biophys Acta 1791:827–835. 10.1016/j.bbalip.2009.05.007 19481615 10.1016/j.bbalip.2009.05.007

[114] Wang X, Hao Y, Wang X et al (2016) A PPARδ-selective antagonist ameliorates IMQ-induced psoriasis-like inflammation in mice. Int Immunopharmacol 40:73–78. 10.1016/j.intimp.2016.08.027 27584056 10.1016/j.intimp.2016.08.027

[115] Warner DR, Liu H, Miller ME et al (2017) Dietary linoleic acid and its oxidized metabolites exacerbate liver injury caused by ethanol via induction of hepatic proinflammatory response in mice. Am J Pathol 187:2232–2245. 10.1016/j.ajpath.2017.06.008 28923202 10.1016/j.ajpath.2017.06.008 PMC5808136

[116] Wecksler AT, Kenyon V, Deschamps JD et al (2008) Substrate specificity changes for human reticulocyte and epithelial 15-lipoxygenases reveal allosteric product regulation. Biochemistry 47:7364–7375. 10.1021/bi800550n 18570379 10.1021/bi800550n PMC2603187

[117] Yamada H, Kikuchi S, Hakozaki M et al (2016) 8-Hydroxyeicosapentaenoic acid decreases plasma and hepatic triglycerides via activation of peroxisome proliferator-activated receptor alpha in high-fat diet-induced obese mice. J Lipids 2016:7498508. 10.1155/2016/7498508 27239345 10.1155/2016/7498508 PMC4864551

[118] Yamada H, Oshiro E, Kikuchi S et al (2014) Hydroxyeicosapentaenoic acids from the Pacific krill show high ligand activities for PPARsS. J Lipid Res 55:895–904. 10.1194/jlr.M047514 24668940 10.1194/jlr.M047514 PMC3995467

[119] Yang Q, Li M, Liu J et al (2023) Intermittent fasting ameliorates neuronal ferroptosis and cognitive impairment in mice after traumatic brain injury. Nutrition 109:111992. 10.1016/j.nut.2023.111992 36871445 10.1016/j.nut.2023.111992

[120] Yang L, Ma C, Zhang L et al (2018) 15-Lipoxygenase-2/15(S)-hydroxyeicosatetraenoic acid regulates cell proliferation and metastasis via the STAT3 pathway in lung adenocarcinoma. Prostaglandins Other Lipid Mediat 138:31–40. 10.1016/j.prostaglandins.2018.07.003 30110652 10.1016/j.prostaglandins.2018.07.003

[121] Yu K, Bayona W, Kallen CB et al (1995) Differential activation of peroxisome proliferator-activated receptors by eicosanoids. J Biol Chem 270:23975–23983. 10.1074/jbc.270.41.23975 7592593 10.1074/jbc.270.41.23975

[122] Zhou QD, Chi X, Lee MS et al (2020) Interferon-mediated reprogramming of membrane cholesterol to evade bacterial toxins. Nat Immunol 21:746–755. 10.1038/s41590-020-0695-4 32514064 10.1038/s41590-020-0695-4 PMC7778040

[123] Zijlstra FJ, Dijk APM, Garrelds IM et al (1992) Species differences in the pattern of eicosanoids produced by inflamed and non-inflamed tissue. Agents Actions 36:C73–C75. 10.1007/BF01996099 1442338

